# Identification of risk- and preventive factors predicting child maltreatment in pregnant women with psychosocial problems

**DOI:** 10.3389/fpsyt.2025.1552740

**Published:** 2025-05-21

**Authors:** Michi Ogawa, Tasuku Hashimoto, Mami Tanaka, Hiroki Ishii, Ryota Seki, Aiko Sato, Atsushi Kimura, Jun Okayama, Mamiko Endo, Naoki Saito, Masaomi Iyo

**Affiliations:** ^1^ Department of Psychiatry, Graduate School of Medicine, Chiba University, Chiba, Japan; ^2^ Department of Psychiatry, Sodegaura-Satsukidai Hospital, Chiba, Japan; ^3^ Department of Psychiatry, International University of Health and Welfare Narita Hospital, Chiba, Japan; ^4^ Department of Psychology, Faculty of Liberal Arts, Teikyo University, Tokyo, Japan; ^5^ Department of Obstetrics and Gynecology, Chiba University Hospital, Chiba, Japan; ^6^ Department of Pediatrics, Graduate School of Medicine, Chiba University, Chiba, Japan; ^7^ Department of Legal Medicine, Graduate School of Medicine, Chiba University, Chiba, Japan

**Keywords:** child maltreatment, family support, perinatal mental health, pregnant women, psychosocial problems

## Abstract

**Introduction:**

Pregnant women with psychosocial problems experience various parenting struggles, which, in the absence of adequate social support, can lead to child maltreatment. This study aimed to identify risk- and preventive factors for child maltreatment among pregnant women with psychosocial problems to facilitate the appropriate allocation of social support.

**Methods:**

This retrospective cohort study analyzed electronic medical records of all consecutive pregnant women with psychosocial difficulties who visited and delivered at Chiba University Hospital, and were registered with the hospital’s child protection team between April 2016 and March 2019. The primary objective was to identify risk- and preventive factors associated with child maltreatment, defined as cases requiring intervention by a child guidance center. Multiple logistic regression analysis was performed to identify the risk factors and preventive factors influencing child maltreatment within the first month postpartum.

**Results:**

A total of 253 pregnant women were included in the analysis, of whom 54 were reported for child maltreatment. Maternal grandmother’s support (OR: 7.85, 95%CI [3.47–17.77]) and other family members’ support (OR: 3.57, 95%CI [1.51–8.41]) were identified as preventive factors. Maternal mental illness (OR: 0.40, 95%CI [0.18–0.89]) was identified as a risk factor. Additionally, the higher the mother’s age (OR: 1.08, 95%CI [1.02–1.14]), the lower the incidence of child maltreatment, indicating that adolescent pregnancy is also a risk factor.

**Conclusions:**

Family support, particularly from the maternal grandmother, plays a crucial role in enhancing parenting skills of women with psychosocial difficulties. Assessing these factors and integrating them into public support initiatives could contribute to the prevention of child maltreatment.

## 1 Introduction

Pregnant women with psychosocial problems—such as mental illness, poverty, a history of child abuse, and intimate partner violence (IPV)—are at an increased risk of experiencing postpartum difficulties related to mental health and parenting (1–3). If left unaddressed, these issues can lead to child maltreatment, underscoring the need for early intervention and social support. Given that child maltreatment is a significant health concern, there is a growing emphasis on developing preventive measures, particularly during the perinatal period. To ensure the effective allocation of social support, numerous population-based studies have sought to identify risk factors for child abuse (4–6). Several factors have been associated with an increased risk of child maltreatment, including adolescent pregnancy, low maternal education level, having two or more children, history of artificial abortion, IPV, and smoking during pregnancy (7–11). Early assessment during pregnancy is particularly crucial, as fatal cases of child maltreatment often occur within the first year of life (12). However, relatively few studies have examined risk factors over time beginning with pregnancy.

While research on risk factors has been widely conducted, the need for research on maternal preventive factors has also been advocated. In particular, family support has been reported to positively impact maternal and child outcomes during pregnancy and the postpartum period. For instance, support from family members during pregnancy can reduce maternal anxiety and the incidence of postpartum depression (13, 14). Moreover, women who receive emotional support from their spouse, family members, and even social networks during pregnancy are less likely to experience perinatal complications such as premature birth (15). Further, the functional support of maternal grandmothers during the perinatal period greatly enhances parenting ability in women with psychosocial problems (16). This suggests that family members’ functional support for pregnant women is valuable not only during pregnancy but also during the postpartum period. The most severe consequence of child maltreatment is fatality. The government bureaus dealing with child health in USA, Canada, and Japan consistently report that infants under one year of age account for highest proportion of child maltreatments fatalities among children under 18 years (17–19). In particular, data from the Children and Families Agency of Japan indicate that newborns less than one month old (i.e., 0-month-old infants) have the highest fatality rate due to child maltreatment among all infants under one years of age. Given this background, the authors hypothesized that the quality and amount of support from family would have the most significant impact on the parenting ability of pregnant women with psychosocial problems. This retrospective cohort study aimed to identify both risk- and preventive factors for child maltreatment in pregnant women with psychosocial problems. Identifying these factors prior to childbirth could enable perinatal care and mental health professionals to establish comprehensive child maltreatment prevention teams prior to delivery. Furthermore, in the context of limited human and financial resources, identifying high- risk cases would allow multidisciplinary support teams to prioritize pregnant women who require urgent intervention. By appropriately allocating resources, such efforts could enhance child protection from the antenatal period onward.

## 2 Methods

### 2.1 Study design and participants

This retrospective cohort study utilized electronic medical records of all consecutive pregnant women with psychosocial problems who met the following inclusion criteria: 1) they continuously visited and delivered at the department of maternal-fetal medicine at Chiba University Hospital in Japan between April 2016 and March 2019; and 2) they received intervention from the hospital’s child protection team (CPT). During the study period, a total of 2,558 deliveries occurred at the hospital. The CPT consolidates information on children who may be suffering from child maltreatment and collaborates with external agencies such as child guidance centers. All pregnant women have the opportunity for a screening interview with an obstetrician or midwife, who administers a clinical interview and questionnaire regarding their medical and sociodemographic information. Women identified with psychosocial difficulties during the screening are eligible for CPT intervention. The CPT comprises a multidisciplinary team, including pediatricians, obstetricians, gynecologists, psychiatrists, nurses, midwives, social workers, and office staff. Participants were excluded if they:1) changed hospitals prior to delivery, or 2) delivered a stillborn baby.

### 2.2 Data collection

The participants’ data, originally collected between April 2016 and March 2019, were obtained from the electronic medical records of Chiba University Hospital. The information was obtained on sociodemographic characteristics, birth history (primipara or multipara), marital status and family structure, medical information such as medical complications and psychiatric disease. Obstetric information such as pregnancy complications and fetal abnormalities, serious economic deprivation such as living on welfare support, caregiving capacity of their families, and child guidance center involvement. Sociodemographic characteristics included age and medical insurance. Data were tracked from the first pregnancy visit through one month postpartum.

### 2.3 Defining child maltreatment

In Japan, the child guidance center is the primary institution that handles cases of child abuse. Child guidance centers offer counseling on all matters related to the development of children and youth, aged 0–17, including children’s rights. Although the role of the child guidance center varies, the most important function is to respond to and protect children when abuse is recognized. The authors, therefore, regarded the intervention of a child guidance center as an indicator of child maltreatment in this study.

### 2.4 Primary outcome measures

The primary outcome measures were the risk and preventive factors for child maltreatment within the first month postpartum in pregnant women with psychosocial problems, including primiparas and multiparas.

### 2.5 Measures

Demographic variables included age, birth history, and economic deprivation (e.g., reliance on welfare support). The dependent variable in this study was child maltreatment, and the independent variables included maternal age, marital status, maternal mental illness, physical and obstetric complications, support from the maternal grandmother, and support from other family members.

### 2.6 Statistical analyses

All statistical analyses were conducted utilizing SPSS version 28 (IBM Corporation, Armonk, NY, USA). Multiple logistic regression analyses were conducted to identify significant predictors of the child guidance center involvement, to calculate odds ratios, and to estimate the strength of the association, if present. To enhance interpretability, the cases with reported child maltreatment were coded as “0,” while the ones without reported child maltreatment were coded as “1.” Consequently, in the figures, an OR < 1 indicates an increased likelihood of child maltreatment, whereas an OR > 1 suggests a reduced likelihood. Explanatory variables were defined as potential risk- and preventive factors associated with child maltreatment. A 95% confidence interval (CI) was utilized to estimate the precision of the odds ratios, and a significant level of *p* < 0.05 was applied.

### 2.7 Ethical considerations

This study was approved by the Ethics Committee of Chiba University Graduate School of Medicine (ID 3498). Given the retrospective nature of the study and the use of anonymized data, the requirement for written informed consent was waived by the committee. To ensure transparency, the study research proposal was disclosed on the Chiba University website using an opt-out approach. This study was conducted in accordance with the principles outlined in the Declaration of Helsinki.

## 3 Results

### 3.1 Participant characteristics

Among the 253 families surveyed, accounting for 9.9% of all deliveries during this study period, 54 families experienced intervention from the child guidance center. The mean age of the pregnant women was 32.75 years with a standard deviation of ±7.08 years. **Table 1** exhibits the participants’ age, childbirth history, marital status, mental illness, physical and obstetric complications, economic deprivation such as living on welfare support, and family support. Additionally, **Table 2** provides details on mental illness, as well as physical and obstetric complications.

**Table 1:** Distribution of patients according to their sociodemographic characteristics (N=253).

Characteristics	No Reported Child Maltreatment (N = 199)		Reported Child Maltreatment (N = 54)	
Age				
Mean age, years (SD)	33.3	± 7.00	30.7	± 6.94
Range of age (min-max)	16-55		17-44	
Under 20 years old N (%)	6	(3.02)	2	(3.70)
Birth history N (%)				
Primipara	114	(57.29)	31	(57.41)
Multipara	85	(42.71)	23	(42.59)
Marital status N (%)				
Single	41	(20.60)	24	(44.44)
Married	158	(79.40)	30	(55.56)
Complications N (%)				
Mental illness	102	(51.26)	33	(61.11)
Physical and obstetric complications	158	(79.40)	40	(74.07)
Economic problems (i.e., receiving welfare public assistance) N (%)	9	(4.52)	7	(12.96)
Support from family members N (%)				
Maternal grandmother	130	(65.33)	18	(33.33)
Other family members	156	(78.39)	32	(59.26)

**Table 2:** Detailed information on mental illness, physical and obstetric complications.

Complications N (%)	No Reported Child Maltreatment (N = 199)		Reported Child Maltreatment (N = 54)	
**Mental illness**	102	(51.26)	33	(61.11)
Mental and behavioral disorders due to psychoactive substance use	3	(1.51)	5	(9.26)
Schizophrenia, schizotypal and delusional disorders	13	(6.53)	7	(12.96)
Mood [affective] disorders	55	(27.64)	11	(20.37)
Neurotic, stress-related and somatoform disorders	21	(10.55)	3	(5.56)
Behavioral syndromes associated with physiological disturbances and physical factors	7	(3.52)	2	(3.70)
Disorders of adult personality and behavior	2	(1.01)	1	(1.85)
Mental retardation	1	(0.50)	4	(7.41)
Physical and obstetric complications				
Physical complications				
Infectious diseases	11	(5.53)	4	(7.41)
Endocrine and metabolic diseases before pregnancy	26	(13.07)	1	(1.85)
Diseases of central nervous system	16	(8.04)	1	(1.85)
Diseases of circulatory system	6	(3.02)	0	(0.00)
Connective tissue diseases	4	(2.01)	0	(0.00)
Diseases of the digestive system	4	(2.01)	1	(1.85)
Diseases of the genitourinary system	17	(8.54)	1	(1.85)
Others	8	(4.02)	1	(1.85)
Obstetric complications				
Abnormalities during pregnancy				
Threatened premature labor	13	(6.53)	3	(5.56)
Blood type incompatibility	4	(2.01)	3	(5.56)
Gestational diabetes mellitus	39	(19.60)	12	(22.22)
Congenital anomalies of the uterus	2	(1.01)	0	(0.00)
Premature rupture of the membranes	15	(7.54)	1	(1.85)
Amniotic fluid abnormalities	5	(2.51)	1	(1.85)
Placental abnormalities	8	(4.02)	2	(3.70)
Cervical incompetency	1	(0.50)	2	(3.70)
Hypertensive disorders of pregnancy	11	(5.53)	3	(5.56)
Labor abnormalities	22	(11.06)	2	(3.70)
Fetal malpresentation and malposition	12	(6.03)	4	(7.41)
Uterine atonic bleeding	3	(1.51)	2	(3.70)
Others	4	(2.01)	0	(0.00)
Fetal abnormalities				
Congenital abnormalities including chromosomal abnormalities	13	(6.53)	2	(3.70)
Multifetal pregnancy	3	(1.51)	0	(0.00)
Fetal growth restriction	4	(2.01)	4	(7.41)
Non-reassuring fetal status	4	(2.01)	0	(0.00)
Others	0	(0.00)	0	(0.00)

The group that did not receive the intervention of the child guidance center was predominantly older than the group that received the intervention. Notably, adolescent pregnant women under the age of 20 years accounted for 8 of the 253 cases (3.2%).

### 3.2 Risk factors and predictive factors for child maltreatment

**Figure 1:**
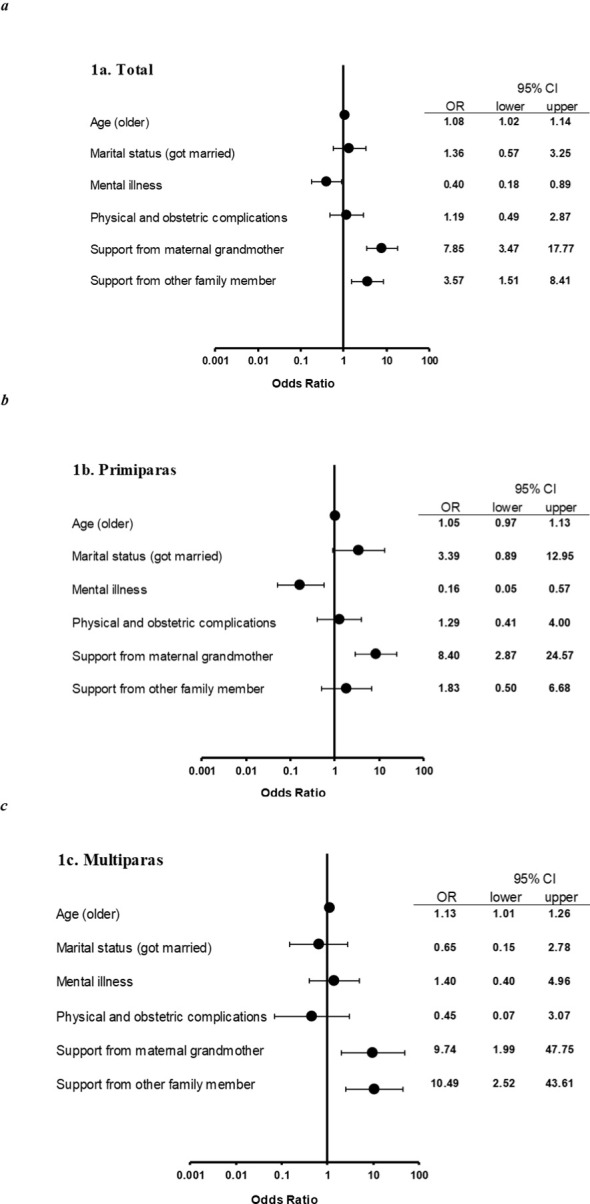
Odds ratios for reported child maltreatment. OR, odds ratio; CI, confidence interval. Odds ratios and 95% CIs for each calculated by multiple logistic analysis of all pregnant women **(a)**, of primiparas **(b)**, of multiparas **(c)**. For all pregnant women **(a)**, maternal mental illness was the risk factor for child maltreatment (OR: 0.40, 95% CI [0.18–0.89]). Older mothers were less likely to be reported child maltreatment (OR: 1.08, 95% CI [1.02–1.14]), indicating that adolescent pregnancy is a risk factor. Preventive factor was support from family (from maternal grandmother: OR: 7.85, 95% CI [3.47–17.77]; from other family member: OR: 3.57, 95% CI [1.51–8.41]). For primiparas **(b)**, maternal mental illness was the risk factor (OR:0.16, 95% CI [0.05–0.57], and support from maternal grandmother was the preventive factor (OR:8.40, 95% CI [2.87–24.57]. For multiparas **(c)**, the risk factor was adolescent pregnancy (OR:1.13, 95% CI [1.01–1.26], and the preventive factors were support from family (from maternal grandmother: OR: 9.74, 95% CI [1.99–47.75]; from other family member: OR: 10.49, 95% CI [2.52–43.61]).

Multiple logistic regression analyses were conducted with child maltreatment as the dependent variable and age, marital status, mental illness, physical and obstetric complications, support from the maternal grandmother, and support from other family members as the independent variables (**Figure 1a**).

Among primipara (**Figure 1b**), pregnant women diagnosed with mental illnesses were more likely to engage in child maltreatment (OR: 0.16, 95% CI [0.05–0.57]), whereas those who received support from their maternal grandmother were less likely to engage in child maltreatment (OR 8.40, 95% CI [2.87–24.57]). For multipara (**Figure 1c**), child maltreatment cases were less frequent if the mother was older (OR 1.13, 95% CI [1.01–1.26]), and pregnant women who received family support were less likely to engage in child maltreatment, whether the support came from their maternal grandmother (OR 9.74, 95% CI [1.99–47.75]) or other family member (OR 10.49, 95% CI [2.52–43.61]).

## 4 Discussion

This study yielded two main findings. First, among pregnant women with psychosocial problems, adolescent pregnancy and maternal mental illness were identified as prenatal predictive risk factors, whereas maternal grandmother’s support and other family members’ functional support were found to be preventive factors for future child maltreatment in the postnatal period. Notably, the maternal grandmother’s support was the strongest preventive factor for child maltreatment. Second, differences were found in risk factors and preventive factors for child maltreatment between multiparas and primiparas. Specifically, psychiatric diseases were a risk factor for child maltreatment in primiparas but not multiparas. Likewise, adolescent pregnancy was a risk factor in multiparas but not primiparas. Among multiparas, support from not only the maternal grandmother but also from other family members also emerged as a preventive factor.

This study exhibits an extremely high odds ratio of parenting support from the maternal grandmother and other family members as a preventive factor for child maltreatment. In prior research, individual factors in the prenatal stage were considered when the public administration assessed pregnant women to identify social support needs. The present findings indicate that individual factors of pregnant women, as well as family contextual factors, need to be considered when assessing the risk for child maltreatment. Less support from family members and the individuals surrounding pregnant women leads to postpartum depression (20), which, in turn, can lead to child maltreatment as it reduces parenting ability. The findings of this study seem to support the hypothesis that the quality and amount of support from family members would have the most significant impact on the parenting ability of pregnant women with psychosocial problems.

The maternal grandmother’s support was a strong preventive factor for child maltreatment among both primiparas and multiparas and can be explained by the following three reasons: (i) Maternal burden is reduced by receiving emotional support from their own mothers (21); (ii) In Japan, the most common form of family support for new mothers comes from the maternal grandmother. It is still a common custom today for a pregnant woman to deliver at her maternal grandmother’s house and remain there for the first 40 days, a practice which is called “Satogaeri Bunben” (22, 23); and (iii) Taking paternity leave is harder than desired in Japan (24). These findings are consistent with our prior study, which indicated that the maternal grandmother’s support reduces the number of institutions providing multidisciplinary support (16). For pregnant women with psychosocial difficulties who have little support from their families, public support agencies should provide intensive support. Moreover, the significant involvement of maternal grandparents in child-rearing, as in Satogaeri bunben mentioned above, is practiced not only in Japan, but also in China and other Asian countries (25, 26). This practice contrasts with that of Western countries, where husbands and partners of pregnant women are heavily involved in child-rearing (27, 28). However, across cultures, the role of grandmothers in postpartum support is increasingly recognized for its benefits to maternal mental health, child-rearing, and newborn well-being (29, 30). This global recognition of grandmothers’ involvement in the perinatal period aligns with the findings of this study, underscoring the need for further research to be conducted on transcultural differences in child-rearing practices and strategies to prevent child maltreatment.

Mental illness prior to delivery and adolescent pregnancy are two predictive risk factors of child maltreatment in the postnatal period identified in this study. The findings of this study on mental illnesses are consistent with those of prior large population-based studies (5, 31, 32). Several plausible explanations exist for why mental illness emerged as a predictive risk factor for child maltreatment. First, a significant proportion of women with mental illness ranging from mild to severe conditions have experience of pregnancy and childbirth (33). Among these, mood disorders—particularly prevalent in this study—are associated with high risk of relapse during the perinatal period (34, 35). Second, neuro- and social cognitive dysfunction leading to impairment of daily living abilities are observed among patients with major psychiatric disorders including major depression, bipolar disorder, and schizophrenia (36–38). Third, mother-infant bonding difficulties among mothers with psychiatric conditions further increase the risk of child maltreatment (39). Considering maternal psychological distress, including depression and anxiety, can lead to mother-infant bonding problems (40), maternal mental illness prior to pregnancy may be a crucial predictive factor for child maltreatment. Finally, women with mental illnesses may have poor relationships with their social circle, lack support, IPV, and have unstable finances (41–43), which may contribute to their reduced ability to raise children. The prevalence of antenatal depression is estimated at approximately 12%, which is a strong risk factor for postpartum depression (44). Therefore, although pregnant women tend to avoid psychiatric treatment (42), particularly with medication, it could be important for those with mental illnesses to provide appropriate psychiatric treatment.

Several prior studies also suggest that adolescent pregnancy is associated with the risk of child maltreatment (7, 11). Regarding adolescent pregnancy and childbirth, it is estimated that 25% of women worldwide experience adolescent pregnancy (45), and that the rate of childbirth in 10–19 year olds is about 11% (46). In Japan, adolescent childbirth accounts for 1.3% of births (47), yet adolescents represent a disproportionately high 17% of mothers involved in fatal child abuse cases (excluding maternal suicides following the murder of their children) (47). Therefore, adolescent pregnancy is an important social problem among low-, middle-, and high- income countries (48). Adolescent pregnancy increases various obstetric complications, such as low birth weight and preterm delivery, as well as a risk factor of severe neonatal conditions (49). In addition, numerous women who become pregnant at a young age drop out of school and lose educational opportunities (49). This makes it more difficult for them to obtain a stable job and family environment in the future (50). In this study, while being a primipara was not a risk factor of child maltreatment, being a multipara was a risk factor. This finding indicates that young women with multipara may experience difficulty with having appropriate parenting practices that lead to child maltreatment. However, there was the possibility that this discrepancy could be due to the small number of pregnant women under the age of 20 years old in this study’s (8 out of 253 cases). Further prospective studies with a large sample of adolescent pregnancy and childbirth are needed to clarify this relationship. In addition, being young and multipara may reflect their unstable environment and the presence of IPV. If some other psychosocial problem coincides with adolescent pregnancy, extensive support interventions should be prioritized.

The study found that the factors affecting parenting ability differ between primiparas and multiparas. Support from the maternal grandmother affected both primiparas and multiparas, whereas support from other family members and being a young mother affected only multiparas. In addition, maternal mental illness affected only primiparas. The finding that family members’ support for multiparas enhances parenting ability can be explained by the following three reasons: (i) In primiparas’ families, husbands do not have childcare experience, but in multiparas’ families, husbands have experience and can therefore play a more active role in childcare. Regarding the role of their husbands or partners as fathers for their newborns and infants, paternity leave is recommended to play a crucial role in supporting their wives and children after deliveries (51, 52). However, an article written by Kahn reports that, as of 2018, the length of paid paternity leave averaged only two weeks in OECD (Organization for Economic Co-operation and Development) countries (51). Notably, while two weeks of paternity leave is sufficient to reduce paternal postpartum depression risk, it is insufficient to mitigate maternal postpartum depression risk (52); (ii) Multiparas’ families also need considerable manpower to raise two or more children. This is indicated by the increased risk of maltreatment in families with three or more children (8, 9); and (iii) As the age of the maternal grandmother increases in multiparas’ families, the assistance of other family members becomes increasingly important. Additionally, during the perinatal periods, women living with parents-in-law may be a risk factor for postpartum depression—assessed by utilizing the Edinburgh Postnatal Depression Scale (53). Therefore, perinatal care and mental health professionals must conduct comprehensive and timely risk assessments to ensure adequate family for vulnerable mothers (54). As noted above, it is likely that being young and multiparous reflects other risk factors, such as economic instability and IPV. Finally, the reason why maternal mental illness was a risk factor for primiparas but not multiparas remains unclear. This may be due to multiparas having more experience in child-rearing, countering the effects of mental illness. Understanding these differences between the characteristics of primiparas and multiparas will enable a more accurate assessment.

### 4.1 Limitations

This study has several limitations. First, the retrospective cohort design resulted in missing information. For instance, detailed financial information was unavailable, except for cases of significant deprivation, such as receiving public financial assistance. The extent to which each family member provided support is also unknown. In postpartum women with husbands or partners, the duration and intensity of paternal leave may have influenced parenting dynamics, but this information was not collected. Similarly, for single mothers, the quality of their relationships with parents, siblings, and other relatives likely impacted their ability to receive childcare support, but this factor was not systematically assessed. Furthermore, the retrospective nature of the study limited the evaluation of child maltreatment. While intervention by a child guidance center served as a promising indicator for child maltreatment, the types and severities of child maltreatment—such as sexual, physical, or psychological abuse, as well as neglect—were not identified. Likewise, this study’s retrospective design, based on medical records, may have missed eligible women who declined CPT intervention. Second, the data were based on midwives’ interviews with patients; therefore, information was omitted if it was not reported by the patient. Third, untreated psychiatric disorders may not have been detected as psychiatrists and psychometric properties or structured interviews for diagnosing mental and behavioral disorders were not available to assess psychiatric symptoms during pregnancy. Fourth, child maltreatment in this study was measured by the intervention of a child guidance center within one month post-delivery, which does not equate to child maltreatment, and the number of cases of child maltreatment discovered at one month postpartum is unknown. Regarding the duration of the survey, longer follow-up studies are required to identify long-term risk- and preventive factors associated with child maltreatment beyond the immediate postpartum phase. Finally, the sample size was relatively small and potentially biased compared to the general population. To establish more robust prenatal predictors of child maltreatment, larger multicenter studies are required. Regarding the bias, the present data was obtained from a single institute, Chiba University Hospital, which specializes in managing pregnant women with severe physical and mental illnesses as well as psychosocial difficulties. Consequently, hospital-based CPTs were actively involved from the perinatal period, not only in suspected cases of abuse but also in cases involving psychosocially vulnerable pregnant women, such as single mothers, those facing financial difficulties, lack of childcare support, and mental or physical health conditions. Although retrospective studies remain important in the field of child maltreatment, further prospective cohort studies with larger samples are required to address these limitations.

## 5 Conclusion

This study demonstrates that the importance of family support, especially from the maternal grandmother, improves the parenting skills among women with psychosocial difficulties. The study also suggests that the previously identified risk factors have different effects on primiparas and multiparas. Assessing risk factors and preventive factors from the antenatal period can help provide more intensive child maltreatment countermeasures, especially when public support resources are limited. Future studies should continue to explore the complex relationships between prenatal psychosocial factors, family support, and child maltreatment to inform the development of effective prevention and intervention strategies. In particular, studies exploring the role of extended family support during the perinatal period and the development of specific interventions—such as multidisciplinary or inter-agency collaborative approaches to perinatal mental health—are highly needed to reduce the risk of child maltreatment.

## Data Availability

The raw data supporting the conclusions of this article will be made available by the authors, without undue reservation.
